# Impact of Plasmonic Nanoparticles on Poikilocytosis and Microrheological Properties of Erythrocytes

**DOI:** 10.3390/pharmaceutics15041046

**Published:** 2023-03-23

**Authors:** Tatiana Avsievich, Ruixue Zhu, Alexey P. Popov, Alexander Yatskovskiy, Anton A. Popov, Gleb Tikhonowsky, Andrei I. Pastukhov, Sergei Klimentov, Alexander Bykov, Andrei Kabashin, Igor Meglinski

**Affiliations:** 1 Optoelectronics and Measurement Techniques, University of Oulu, 90570 Oulu, Finland; 2 VTT Technical Research Centre of Finland, Kaitovayla 1, 90590 Oulu, Finland; 3 Department of Histology, Cytology and Embryology, Institute of Clinical Medicine N.V. Sklifosovsky, I.M. Sechenov First Moscow State Medical University, Trubetskaya Street 8, 119991 Moscow, Russia; 4 Institute of Engineering Physics for Biomedicine (PhysBio), National Research Nuclear University (MEPhI), Kashirskoe Shosse, 31, 115409 Moscow, Russia; 5 CNRS, LP3, Aix-Marseille University, 163 Av. de Luminy, 13009 Marseille, France; 6 College of Engineering and Physical Sciences, Aston University, Birmingham B4 7ET, UK

**Keywords:** plasmonic nanoparticles, red blood cells, microrheology, aggregation, cell–cell interactions, poikilocytosis, optical manipulation, microscopy

## Abstract

Plasmonic nanoparticles (NP) possess great potential in photothermal therapy and diagnostics. However, novel NP require a detailed examination for potential toxicity and peculiarities of interaction with cells. Red blood cells (RBC) are important for NP distribution and the development of hybrid RBC-NP delivery systems. This research explored RBC alterations induced by noble (Au and Ag) and nitride-based (TiN and ZrN) laser-synthesized plasmonic NP. Optical tweezers and conventional microscopy modalities indicated the effects arising at non-hemolytic levels, such as RBC poikilocytosis, and alterations in RBC microrheological parameters, elasticity and intercellular interactions. Aggregation and deformability significantly decreased for echinocytes independently of NP type, while for intact RBC, all NP except Ag NP increased the interaction forces but had no effect on RBC deformability. RBC poikilocytosis promoted by NP at concentration 50 μg mL${}^{-1}$ was more pronounced for Au and Ag NP, compared to TiN and ZrN NP. Nitride-based NP demonstrated better biocompatibility towards RBC and higher photothermal efficiency than their noble metal counterparts.

## 1. Introduction

Plasmonic nanoparticles (NP) present one of the most important classes of nanomaterials for biomedical usage [1,2,3]. Owing to optical excitations of collective free electron oscillations (plasmons), these NP can exhibit a strong absorption band in optical spectra with the absorption cross-section exceeding that of conventional absorbing dyes by orders of magnitude [3]. This makes them excellent candidates as sensitizers of photo-induced therapy [2,3] and contrast agents in photoacoustic imaging [2,4], while a local enhancement in electric field promising their use as substrates for SERS-based bioidentification [5,6]. Gold (Au) and silver (Ag) nanostructures present conventional plasmonic nanomaterials and are widely used across a variety of applications. Both Au and Ag NP provide strong SERS signals [7], while Ag nanoparticles can serve as efficient antibacterial agents [8]. Au nanostructures are considered as excellent candidates for phototherapy, but one has to design complex structures (core-shells, nanorods) to shift the plasmonic peak (originally generated around 520–550 nm) toward the window of relative tissue transparency (650–900 nm) [2,3]. The plasmonic mismatch problem of conventional plasmonic materials can also be solved by the employment of alternative titanium nitride (TiN) and zirconium nitride (ZrN) NP, which generate plasmonic bands centred around 700 nm and 670 nm, respectively [9,10].

Typically, regardless of the route of entry into the human body NP are transported through the blood circulation system interacting with blood components [11,12]. Hence, hemocompatibility is a primary requirement for NP safety evaluation [13]. Red blood cells (RBC), being the most abundant cellular component of blood (40–45% volume percentage), are significant contributors to blood properties. The unique biconcave shape and high deformability of RBC enable them to squeeze through the narrowest capillaries to perform the key function of gas exchange in tissues [14]. RBC are commonly used for NP testing due to their availability and structural simplicity [15] and are actively investigated as a natural drug delivery system (DDS) [12,16], underlying the need for NP evaluation at a cellular level [17]. Many NP were found to influence RBC: fullerene NP soften RBC membrane [18], nanodiamonds may induce RBC aggregation [19], polystyrene NP adsorbed on RBC membrane or incorporated into the RBC can affect cellular deformability [17]. Exposure to NP can lead to impaired RBC properties, causing hemolysis, poikilocytosis (an increase in abnormal-shaped RBC), and alterations of deformability and aggregation. These changes can further result in complications of blood microrheology, ultimately leading to thrombosis [20,21,22].

NP prepared via pulsed laser ablation in liquids (LAL) present a novel class of ultrapure nanomaterials for biomedical applications. This relatively new method is based on a natural production of nanoclusters under the action of laser radiation on a solid target in a liquid ambient (deionized water, ethanol, etc.), followed by their release into liquid ambience to form a colloidal NP solution [23,24,25]. The advantage of this approach is related to the possibility of performing the synthesis in an ultraclean environment in the absence of toxic by-products typical for conventional chemical synthesis. Using this approach, a series of novel nanomaterials, including plasmonic Au NP [26,27,28], Ag NP [29] and TiN NP [30,31,32], were recently synthesized to explore their use in biomedical tasks. Laser-synthesized TiN NP can provide local hyperthermia under photoexcitation in the window of relative tissue transparency [30,31] and be used as contrast agents in photoacoustic imaging [32], while Au NP can be employed as electrocatalyzers of glucose oxidation [27] and SERS probes for identification of biological molecules [33,34].

In the present study, the influence of plasmonic NP synthesized by LAL on RBC microrheology and poikilocytosis was studied on RBC in vitro. We characterized the toxicity of Ag, Au, TiN and ZrN NPs towards RBC by assessing their hemolytic activity with optical spectroscopy, NP-induced RBC morphological alterations with SEM, and RBC deformability and aggregation in autologous blood plasma via the optical tweezers (OT) method [35]. We also observed a photothermal response of NP to focused infrared (IR) laser radiation leading to hyperthermic destruction of RBC.

## 2. Materials and Methods

### 2.1. Nanoparticles Synthesis and Characterization

Colloidal solutions of plasmonic NP were synthesized by ultrafast (fs) laser ablation of the Au, Ag, and TiN solid targets in deionized water. The target was fixed vertically on the wall of a quartz cuvette filled with 15 mL of ultrapure deionized water (18.2 M ω cm at 25 °C). Radiation from a Yb:KGW laser (1030 nm wavelength, 250 fs pulse duration, up to 30 μJ pulse energy, 100 kHz repetition rate, TETA 10 model, Avesta, Moscow, Russia) was focused by a 100 mm F-theta lens on the surface of the target through a side wall of the ablation vessel. The laser beam was scanned over a 20 × 10 mm${}^{2}$ area on the surface of the target, with 4000 mm/s speed with the help of a galvanometric scanner (LScanH-10-1030, AtekoTM, Moscow, Russia). The scanning was performed to avoid hole drilling in the target and to prevent the scattering of laser pulses on cavitation bubbles, which ultimately resulted in a significant NP yield increase. The thickness of the liquid layer along the incident laser beam was 4 mm. The interaction of extremely powerful fs laser pulses with liquid ambient was accompanied by energy-dependent self-focusing of the laser beam. The power of the laser pulses was 120 MW, which significantly exceeds the self-focusing threshold of water at 1030 nm (14.8 MW). Therefore, to take into account shifts of the focal plane due to the self-focusing effect, the target-lens distance was adjusted to obtain the maximum NP yield of the ablation process, measured by weighing the target before and after the ablation. Concentrations of the obtained colloidal solutions were 150 μg mL${}^{-1}$ for Ag NP, 100 μg mL${}^{-1}$ for TiN NP and 200 μg mL${}^{-1}$ for Au NP. The duration of each experiment was 15 min. To produce ZrN-based NP, the target was placed vertically in a quartz vessel filled with 25 mL of deionized water. A laser beam (Amplitude Systems, 1025 nm, 490 fs, 50 kHz) was focused on the surface of the target by a 75 mm convex lens. The energy of the incident beam was preliminarily attenuated by a half-wave plate and Brewster polarizer to 30 μJ per pulse. The calculated concentration of NP in the solution was about 110 μg mL${}^{-1}$ and was estimated by measuring the mass of the target before and after the process of ablation.

Morphology, structure and size distributions of obtained plasmonic NP were characterized by transmission electron microscopy (TEM) using a JEM-3010 microscope (JEOL, Peabody, MA, USA) at an acceleration voltage of 300 kV or scanning electron microscopy (SEM) using a MAIA 3 microscope (Tescan, Brno, Czech Republic) operating at 20 kV accelerating voltage. Samples for the electron microscopy were prepared by dropping 10 μL of the NP suspension onto a formvar-coated copper grid (200 mesh, Oxford Instruments, Oxford, UK) or a cleaned crystalline silicon substrate (for SEM), with subsequent drying at ambient conditions. The data from the TEM and SEM images were analyzed by Fiji ImageJ software (National Institutes of Health, USA). The optical extinction spectra were recorded using a UV-Vis spectrometer (UV-2600, Shimadzu, Tokyo, Japan for ZrN NP or PSI-MC 2, SOL Instruments, Minsk, Belarus) in optical glass cuvettes with a path length of 10 mm. Stock NP water solutions were centrifuged at 17,000× *g* (Micro Star 17, VWR, Radnor, PA, USA) for 35 min, solvents were removed and substituted with Dulbecco’s Phosphate Buffered Saline (DPBS, Sigma Aldrich, pH 7.4, St. Louis, MO, USA). The estimation of PdI (polydispersity index) and zeta potential of of NP colloids was performed using Malvern Zetasizer Nano ZS (Malvern Instruments, Malvern, UK). NP absorbance spectra were also examined for NP dissolved in blood plasma with a spectrophotometer (Optronic Laboratories, Orlando, FL, USA) in the transmission mode.

### 2.2. Blood Samples

Whole blood specimens were obtained from two healthy volunteers by venipuncture at the Nordlab clinic (Oulu, Finland) with oral consent and ethical permission (Finnish Red Cross, No. 6095, 7/2021). Blood was collected in BD Vacutainer tubes containing EDTA (ethylenediaminetetraacetic acid) as an anticoagulant. Whole blood was centrifuged (CompactStar CS4, VWR, Radnor, PA, USA) at 2000× *g* (4500 RPM) for 10 min to remove formed elements. The supernatant was gently aspirated and the process was repeated at 4000× *g* (6500 RPM) to obtain platelet-free plasma. Healthy human RBC were freshly collected from fingertip-prick blood drops prior to each series of experiments. Blood (20 μL) was washed twice with DPBS and centrifuged at 3000× *g* for 10 min. NP dissolved in DPBS were prone to agglomeration, therefore prior to incubation with RBC, NP were sonicated for 1–2 min with a probe ultrasonic homogenizer (Bandelin Sonoplus HD 2070.2, Berlin, Germany) to obtain homogenous suspensions. Washed RBC were incubated with NP for 1 h at 1% hematocrit (1% Ht) to keep the constant RBC/NP ratio for all experiments. After incubation samples were centrifuged at 2000× *g* for 5 min. Supernatant was used for hemolytic activity examination. Intact RBC formed a dark pellet on the bottom of the tube. Sedimented RBC were diluted in plasma at 3% hematocrit for optical microscopy observation and at 0.5% hematocrit for OT. The sample preparation and experiments were conducted at room temperature 23 °C.

### 2.3. Hemolytic Activity

RBC were dispersed in DPBS NP solutions of 100, 50 and 10 μg mL${}^{-1}$ at 1% Ht. RBC incubated in distilled water (DI) and DPBS (without NP) served as positive (+) and negative (−) controls, respectively. All mixtures were gently vortexed and incubated at room temperature (23 °C) for 1 h. After incubation, samples were centrifuged at 2000× *g* for 5 min, and 500 μL of supernatant was transferred into a quartz cuvette to measure the absorbance spectra (400–800 nm) of hemoglobin from lysed RBC with a spectrophotometer (Optronic Laboratories, Orlando, FL, USA) in the transmission mode. Among the 3 hemoglobin characteristic peaks at 415, 541 and 577 nm, the one at 541 nm was taken for analysis. Hemolysis percentages toward RBC in the samples were calculated using the following formula:

(1) $$Hemolysis\left(\%\right)=\frac{\left(Abs(sample)-Abs(-)\right)}{\left(Abs(+)-Abs(-)\right)}\times 100\%,$$

where Abs(sample) is the absorbance of the treated sample, Abs(+) and Abs(−) is the absorbance of the positive and negative control, correspondingly. Data presented as the mean ± standard deviation (SD) of three independent experiments. One-way ANOVA with post hoc Dunnett’s test (no matching or pairing) was performed in GraphPad Prism (GraphPad Software, San Diego, CA, USA). Statistical significance is presented for each sample against the control (* *p* < 0.05).

### 2.4. Optical Microscopy

RBC incubated with NP in DPBS were resuspended in autologous blood plasma at 1% Ht. Samples were gently vortexed and transferred into a measurement chamber (15 μL) made from a microscopic cover glass attached to a microscopic slide with double-sided tape. Opened edges were sealed with Vaseline to prevent drifts inside the sample due to drying. The sample was immediately placed for microscopic examination (Nikon Eclipse LV100 Upright Microscope, Tokyo, Japan) in the bright-field mode. Images were captured by supplementary NIS-Elements Advanced Research Imaging Software. The automated timelapse was recorded for each sample within 2 h with an interval of 2 or 5 min from the moment when all RBC were settled on the bottom of the cuvette. Each image was then analyzed to calculate the percentage of image area occupied by RBC. Initially, most RBC are oriented face-front to the camera, but then over time they flip on the edge while forming the aggregates, hence the whole area occupied by RBC decreases. Monochrome images (1600×1200 px) were then analyzed with ImageJ software (National Institutes of Health, Bethesda, MA, USA). The threshold (“Mean” method) was set to identify RBC in the stack of the images, and the image was converted to a binary image. Then RBC were analyzed automatically (command “Analyze particles; the minimum detectable particle was set to an area of one RBC (5280 px${}^{2}$) or the area of the edge-on oriented RBC 20 μm${}^{2}$. About 700–800 cells were analyzed per sample. Results displayed the relative area of RBC occupied by RBC in percentages.

### 2.5. Optical Tweezers

A double-channel OT setup [19,36,37] was used to measure the RBC aggregation force and deformability in autologous plasma at 0.5%Ht. Two optical traps were formed by orthogonally polarized continuous-wave laser beams from a single-mode Nd:YAG infrared laser ILML3IF-300, λ = 1064 nm, 350 mW (Leadlight Technology, Taoyuan, Taiwan) and a 100 (*NA* = 1.00) water-immersion objective (Olympus, LumPlanFI, Center Valley, PA, USA). One trap was set to a fixed position, while the second trap was moved by a steerable mirror. Trapped RBC were imaged in a transmission mode with a CMOS camera PL-D722MU-T (Pixelink, Gloucester, ON, Canada). Sample positioning was performed with a micro-resolution translational motorized XY stage 8MTF (Standa, Vilnius, Lithuania). OT calibration was performed using the drag force method described in our previous studies [19,36,37]. To measure the aggregation force between two individual RBC, cells were trapped and lifted at 30 μm height from the bottom of an in-house made measurement chamber (as used for optical microscopy studies described above). RBC were positioned to form a contact area with 40% overlap. The trapping power in both traps was then slowly decreased until RBC started to aggregate. The trapping power at this moment was proportional to the aggregation force, which was then retrieved using the calibration curve. The trapping force is dependent on the RBC shape, therefore calibration curves were obtained for discosytes and distorted RBC shapes—echinocytes. The deformability of RBC was estimated by subjecting the trapped RBC to viscous fluid flow (velocity 100 μm/s). The relative RBC elongation for the control sample and for samples incubated with NP was calculated as:

(2) $$Elongation\left(\%\right)=\frac{L-L_{0}}{L_{0}}\times 100\%,$$

where *L* is the length of the stretched RBC, $L_{0}$ is the initial length of the trapped RBC. Each series of measurements was carried out on 20–30 cells. Experimental data were evaluated by one-way ANOVA with post hoc Tukey’s test. Results are given as the mean ± SD. The values of * *p* < 0.1, ** *p* < 0.05 were considered as significant.

### 2.6. Scanning Electron Microscopy of RBC

RBC incubated with NP for 1 h in DPBS were fixed by adding 1% glutaraldehyde (Merck, Baltimore, MD, USA) for 30 min. After that, the samples were centrifuged at 2000× *g* for 5 min, and the supernatant was discarded. Sedimented RBC were washed with distilled water twice. After platinum sputtering, RBC smears were investigated with Zeiss Sigma FE-SEM (Carl Zeiss, Jenna, Germany) at 5 kV.

## 3. Results

### 3.1. Plasmonic Nanoparticles

**Figure 1:**
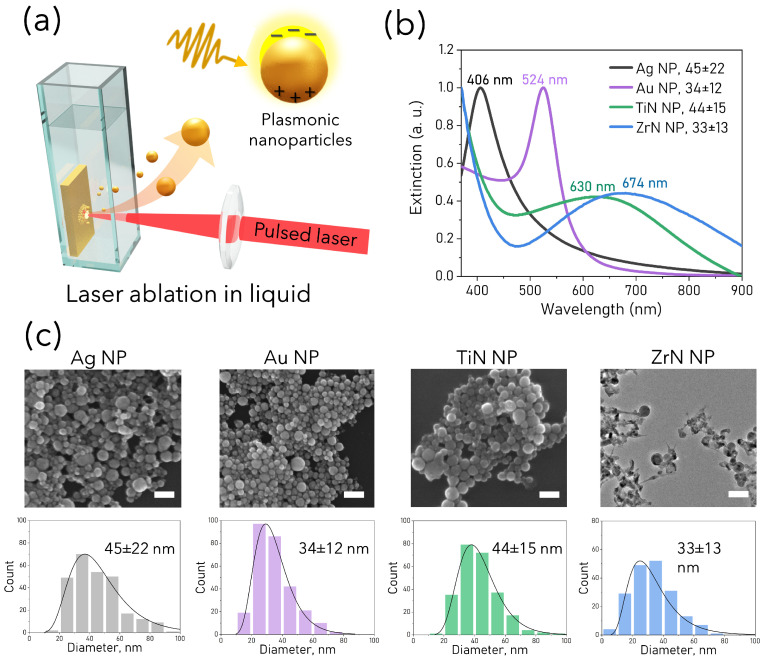
(**a**) Plasmonic NP synthesized by laser ablation in liquid method (LAL). (**b**) Normalized optical extinction spectra of Ag (black), Au (violet), TiN (green) and ZrN (blue) NP. (**c**) SEM images of plasmonic NP synthesized by LAL and their corresponding size distributions: Ag NP, Au NP, TiN NP, ZrN NP. Scale bar: 100 nm.

Laser ablative synthesis resulted in predominantly spherical NP with a mode size of 30–40 nm as shown in Figure 1. Only ZrN NP had a significant fraction of non-spherical nanostructures, and it should be noted that only spherical NP were measured for distribution. Extinction spectra of aqueous colloidal solutions of the synthesized NP are shown in Figure 1b. Characteristic plasmonic peaks of Ag NP at 406 nm, Au NP at 524 nm, ZrN NP at 630 nm and TiN NP at 674 nm. Zeta potential measured for NP in DPBS revealed higher colloidal stability of Ag NP with ζ-potential −28.4±1.6 mV, while Ag, TiN and ZrN NP having originally higher ζ-potential in water (see Supplementary Materials Table S1), in DPBS became less stable with ζ-potential around −20 mV. PdI indices were below 0.5 for NP, indicating relatively narrow size distribution.

### 3.2. Hemolytic Activity of NP

**Figure 2:**
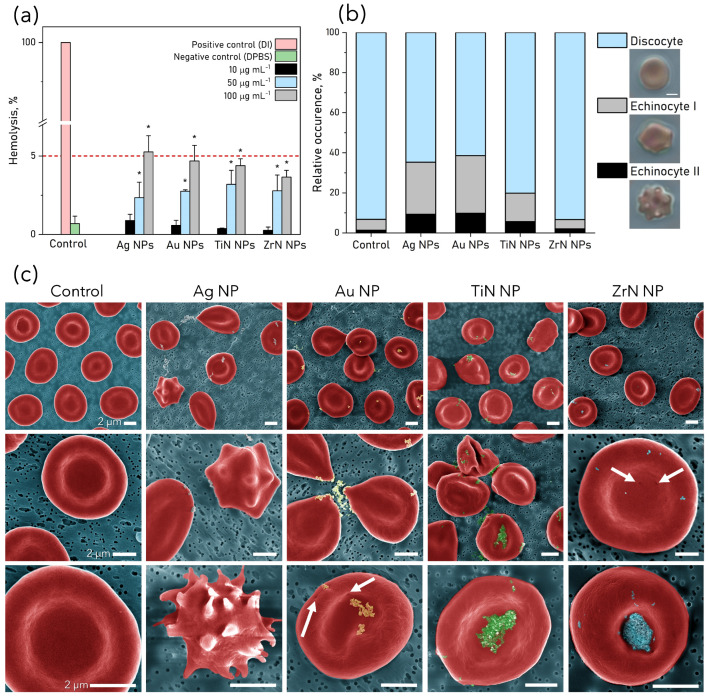
(**a**) In vitro hemolysis test results for RBC (1% hematocrit) exposed to NP for 1 h at concentrations 10, 50 and 100 μg mL${}^{-1}$. The red dashed line indicates the hemolysis threshold value (5%), from which the tested NP are considered hemolytic. RBC in DPBS were used as a negative control, and RBC in DI were used as a positive control. Data are presented as a mean ± SD of three independent experiments and analyzed by one-way ANOVA (post hoc Dunnett’s test). * *p* < 0.05 are significantly different from the negative control. (**b**) The relative occurrence of RBC fractions by morphology: discocyte, echinocyte I and II (EI and EII), in the samples after 1 h incubation with NP and resuspension in plasma at 50 μg mL${}^{-1}$. Scale bar: 3 μm. (**c**) Coloured SEM images of RBC incubated for 1 h with plasmonic NP (50 μg mL${}^{-1}$). Scale bar: 2 μm.

Hemolysis describes the rupturing of RBC and the release of their contents, including hemoglobin, into the extracellular environment. Hemolytic activity of NP was assessed spectrophotometrically, results are presented in Figure 2a. According to ASTM E2524-22 (standard test method for analysis of hemolytic properties of NP) samples exhibiting hemolysis values over 5% correspond to hemolytic samples [38]. The minimal concentration of NP (10 μg mL${}^{-1}$) was not significantly different from the control sample for all NP, 50 μg mL${}^{-1}$ caused insignificant hemolysis. Ag and Au NP were hemolytic at a concentration 100 μg mL${}^{-1}$. NP did not shift the absorbance peaks of hemoglobin.

### 3.3. RBC Poikilocytosis

In total, three main RBC shapes were identified on optical images of the tested samples: a normal discocyte, a mildly perturbed echinocyte I (EI)—an irregularly contoured discocyte with up to five protrusions, and a highly perturbed echinocyte II (EII)—an oval and spherical RBC with many spicules. The relative occurrence of these fractions in each sample is presented in Figure 2b. The percentage of crenated RBC (EI and EII) increased by 5 times in samples treated with Au NP and Ag NP, from 7% in the control sample to 38% and 35% for Au and Ag NP, respectively, a lower number was found for TiN NP 20%, and no change was found for ZrN NP.

The morphology of RBC upon the NP attachment was examined via SEM (Figure 2c). For RBC treated with Ag NP besides the perturbed shapes such as teardrop-like (dacrocytes) and echinocytes, strongly spiked acanthocytes RBC were also found (<1%). The membrane perforations were found in samples treated with Au NP and ZrN NP (shown with arrows on Figure 2c, which could be a result of NP internalization into the cell. Approximately 20% of RBC incubated with Ag and Au NP were prone to exhibit a drop-like shape. More pronounced membrane transformations appeared as NP concentration increased to 100 μg mL${}^{-1}$ (Supplementary Materials, Figure S1).

**Figure 3:**
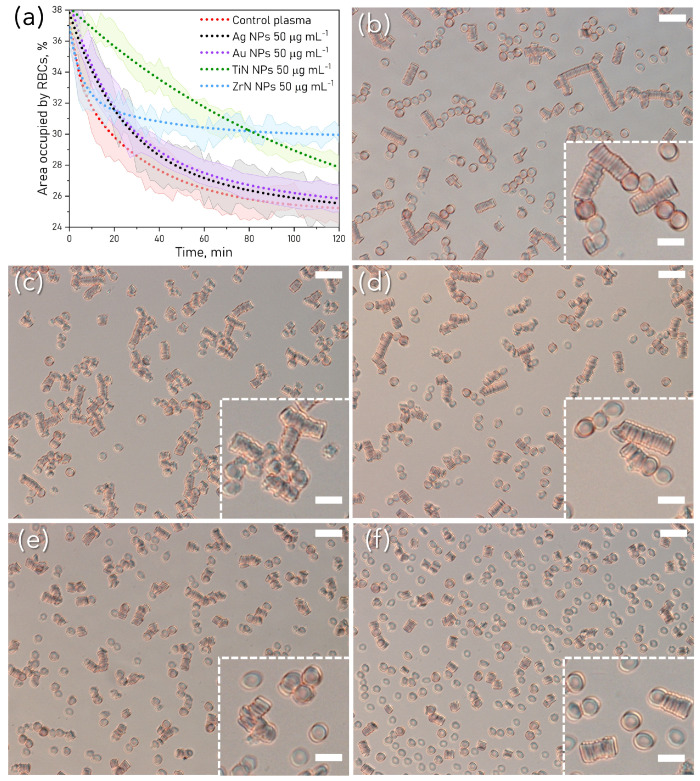
(**a**) Kinetics of RBC aggregation as the change of area occupied by RBC. Dotted lines are average data fitted by one-exponential decay with shaded areas representing the standard deviation. (**b**–**f**) Optical microscopy images of RBC incubated with NP with enlarged insets of aggregates after 2 h in autologous blood plasma: (**b**) control, (**c**) Ag NP, (**d**) Au NP, (**e**) TiN NP, and (**f**) ZrN NP at concentration 50 μg mL${}^{-1}$. Scale: 20 μm (main images), 10 μm (insets).

The area occupied by RBC varied within 10% for the samples; however, this variation did not affect the general trend of exponential decrease, which was found repeatedly for each sample in three independent experiments. The temporal parameters of RBC aggregation were derived from the one-exponential fitting of the kinetics curves (Figure 3a). Within the first minute of observation, RBC were oriented with their face-side parallel to the glass slide of the sample chamber, occupying about 30–40% of the image area. When RBC start to aggregate, this area decreases with time due to the reorientation of RBC from “face-on” to “edge-on” position. The aggregates formed after 2 h in blood plasma in each sample are shown on microscopic images in Figure 3 for control (b), Ag NP (c), Au NP (d), TiN NP (e) and ZrN NP (f). RBC morphological changes caused by NP are shown on bright-field images (enlarged insets). Due to the higher number of single RBC at the beginning, the area occupied by RBC changed more rapidly. Aggregation kinetics retrieved from optical microscopy images revealed the difference between NP-treated samples. The time constants of aggregation kinetics on multi-cellular level for the control (23.8±1.1 min), Ag and Au NP-treated samples were in the same range (29.4±1.4 and 28.3±1 min correspondingly), while the time constant for Zr NP sample was higher (119.34±16.9 min), and significantly lower for TiN NP (13.2±1.3 min) (see Supplementary Materials, Table S2).

### 3.4. RBC Aggregation and Deformability

**Figure 4:**
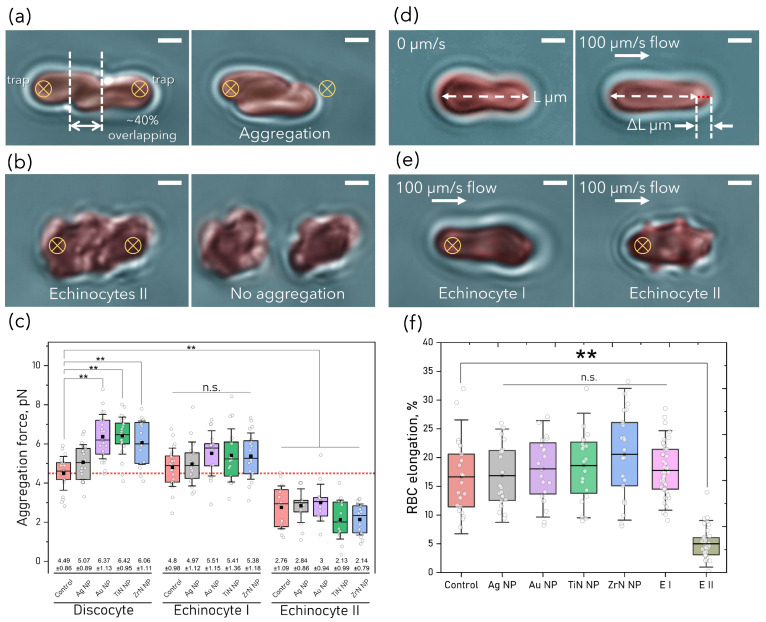
(**a**) OT measurements of RBC aggregation force after treatment with NP (50 μg mL${}^{-1}$) for (**a**) discocytes and (**b**) echinocytes II. RBC relative elongation in fluid flow (100 μm/s) for (**d**) discocyte and (**e**) echinocytes I and II. Scale bar: 2 μm. (**c**) Box plot of RBC aggregation force and (**f**) scatter interval plot of relative RBC elongation in viscous flow 100 μm/s. Data are expressed as mean ± SD, *n* = 20–30, ** *p* < 0.05, ns: non-significant, in one-way ANOVA, followed by Tukey’s post hoc test compared to control. Center lines show the medians; square dots represent the means; outliers and individual data points are represented by dots; box limits indicate the 25th and 75th percentiles; whiskers show SD.

RBC aggregation and deformability were examined at a non-hemolytic concentration 50 μg mL${}^{-1}$. Aggregation force measurements were performed for intact discotic, and for crenated RBC shapes such as EI and EII. The schematic of OT measurements is presented for discocytes and EII on Figure 1a,b. The aggregation force value measured in the control sample without NP was 4.49±0.86 pN. All NP except Ag NP caused an increase in RBC aggregation (Figure 4c). The aggregation force did not change with RBC shape transition into EI; however, EII demonstrated significantly reduced aggregation force compared to the control.

Relative RBC deformation was defined by applying viscous blood plasma flow (100 μm/s) against a trapped RBC for discocytes and for EII (Figure 4d,e). Considering the occurrence of EI and EII, their deformability was also estimated. Interestingly, the behaviour of echinocytic cells was similar in all the NP-treated samples, therefore considering no significant difference among EI and EII of control and NP-treated samples, the elongation data were merged in one group for all NP types (see Figure 4f). There was no statistically significant difference between the values for RBC elongation for normal discocytes and EI RBCs after NP treatment, while for EII RBC elongation was reduced by 70%.

**Figure 5:**
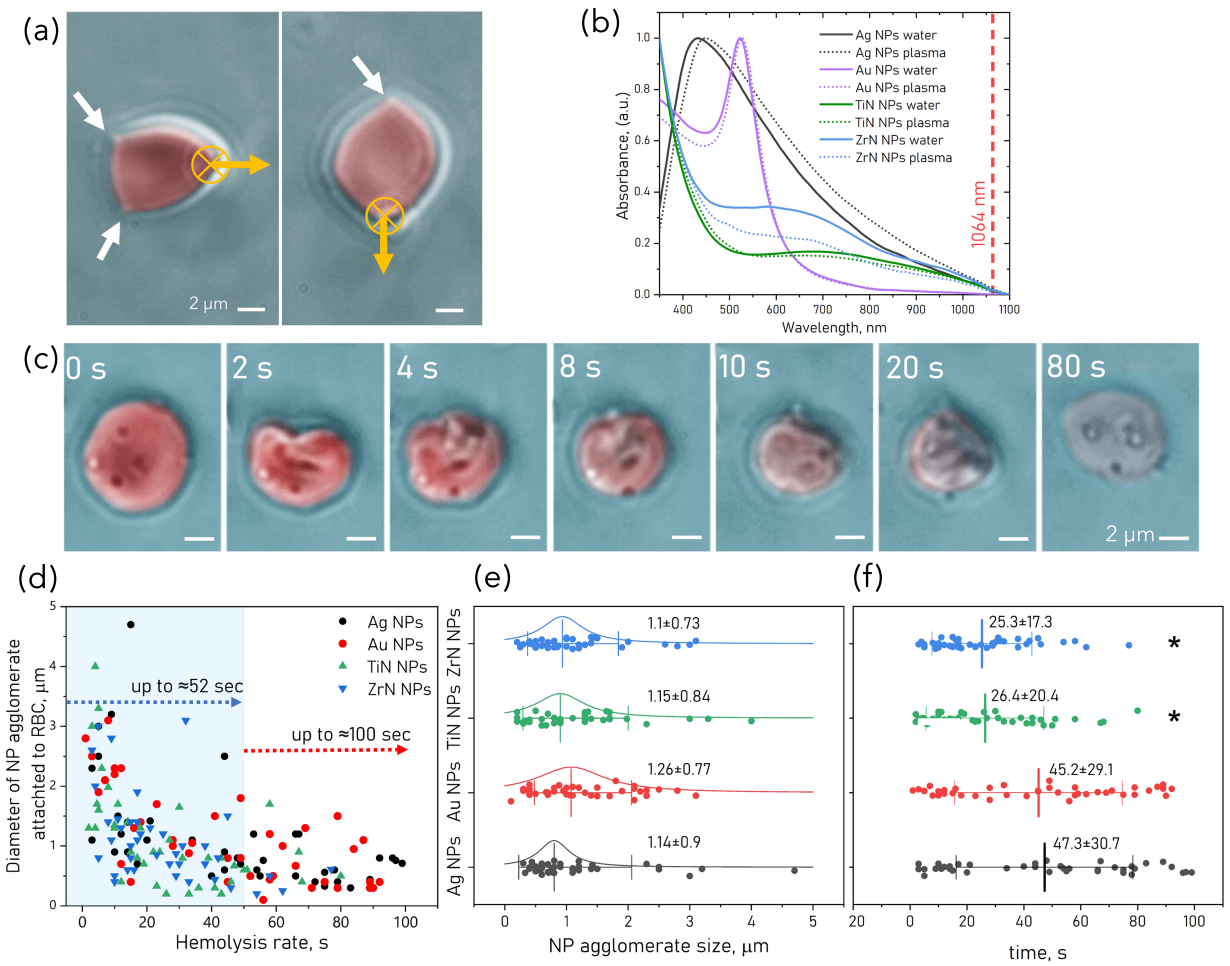
(**a**) RBC treated with ZrN (**left**) and TiN (**right**) NP adhered to the bottom of the measurement chamber. Regions of RBC stuck to the bottom are shown with arrows. Optical trap at maximum power 180 mW (55 pN) was unable to trap RBC. (**b**) Normalized absorption spectra of NP dispersions in water and blood plasma. (**c**) Camera snapshots during 80 s of photothermal destruction of RBC with attached Au NP clusters indicated by arrows in a laser trap (power 40 mW). RBC becomes pale as the optical contrast decreases due to hemolysis. (**d**) Scatter plot of laser trap time exposure vs. size of NP aggregates. The shadowed area indicates the maximum time required to destruct an RBC with attached TiN and ZrN NP. Scatter interval plots (one-way-ANOVA, post hoc Tukey’s test, *n* = 40, comparisons with significant differences are marked for * *p* < 0.05) of mean ± SD for NP agglomerate sizes (**e**) and time of RBC destruction (**f**).

In samples treated with TiN and ZrN NP, many cells strongly adhered to the bottom of the glass measurement chamber while maintaining a discocyte shape, which apparently explained the remarkable difference in aggregation kinetics observed with optical microscopy. Optical trapping of 80% of RBC in these samples was not possible even at the highest power of 180 mW (55 pN). Partial adherence of RBC was visible when some RBC parts could be trapped. Attempting to move such RBC caused its stretching, where adhered parts appeared as protrusions (see Figure 5a).

### 3.5. NP-Induced Hyperthermia of RBC

The absorption spectra of NP dispersions in blood plasma compared to NP spectra in water indicate non-significant shifts of plasmonic peaks (see Figure 5b), indicating that NP preserved the optical properties in the complex biological medium. Still, 16 nm red shift observed for Ag NP could be a result of NP agglomeration in blood plasma. There was no enhanced absorption found at 1064 nm. Tightly focused laser beams of OT give rise to intensities on the order of MW/cm${}^{2}$ at their focus area, which may lead to the consequent heating due to the light absorption. At 100× magnification, NP clusters of size 0.2–3 μm attached to RBC often appeared visible (see Figure 5c). Usually, these RBC cannot be optically trapped. All types of plasmonic NP showed the absorption of 1064 nm irradiation, which was strong enough to cause RBC rupture.Local radiation of the NP attached to RBC by a focused laser beam results in heat release by NP sufficient to induce fast membrane rupture. The laser power density at the focal spot (2 μm) of OT can reach about 2.5 MW/cm${}^{2}$ for the trapping power 40 mW at the beam waist. The time of thermoplasmonic destruction of RBC varies in the range of 1–100 s (Figure 5d), and it largely depends on the applied laser power, the size of NP agglomerates, and the number of aggregates attached to RBC membrane due to the heterogeneous distribution of NP on the RBC surface. An example of RBC destruction resulting in RBC lysis and the formation of the ghost residue of RBC is shown in Figure 5c.

The time required for RBC to burst was almost twice as fast for TiN and ZrN NPs (up to 52 s) compared to longer times (up to 100 s) for Au and Ag NP. The mean sizes of NP agglomerates between the samples were not statistically different (Figure 5e), but hemolysis was reached faster for larger NP agglomerates (Figure 5d). The mean times (Figure 5f) are two-fold shorter for TiN and ZrN NP. Therefore, TiN and ZrN NP have higher plasmonic efficiency compared to their noble counterparts. The plasmonic peaks of TiN NPs and ZrN NP are also red-shifted compared to their counterparts, having an extended peak beyond 800 nm, closer to the trapping wavelength.

## 4. Discussion

LAL technique of NP synthesis offers a set of advantages as a contamination-free, fast and easily adjustable method applicable for various NP types. Since blood is an interface of utmost importance for NP biomedical applications, here we focused on NP-related effects caused by RBC-NP interactions. Recently, high safety and favourable biodistribution of laser-synthesized plasmonic NP was demonstrated in vivo on mice [28,29,31]. In vitro testing of the cellular parameters serves as a first safety assessment step of newly developed laser-synthesized plasmonic NP for biomedical applications.

NP demonstrated concentration-dependent hemolytic activity, with a lower hemolysis rate of TiN and ZrN NP compared to Ag and Au NP. Hemolytic 5% threshold indicating severe RBC damage was observed for all NP at concentrations exceeding 100 μg mL${}^{-1}$. Our results align with those from a previous study that showed TiN NP to be highly biocompatible towards RBC in vitro and in vivo [31] at concentrations up to 100 μg mL${}^{-1}$. The absence of hemolysis, however, does not exclude other adverse effects, such as RBC poikilocytosis, deformability or aggregation.

In the samples treated with 50 μg mL${}^{-1}$ NP, the fraction of morphologically altered EI and EII was increased in RBC population. Each detected fraction contributes to the total aggregation behaviour of RBC ensembles observed by optical microscopy. Single RBC analysis with OT is limited by the lower number of samples, which are represented by “trappable” RBC in our measurements. In turn, this allows us to measure the interaction force between the three types of RBC found in each sample (discocyte, EI, EII). The kinetics of RBC ensemble aggregation were found to be significantly different for samples treated with TiN and ZrN NP, the main reason for this difference identified with OT was a result of strong RBC adherence to the glass bottom of the chamber. A faster RBC aggregation rate was observed in ZrN NP-treated sample, while TiN NP slowed down the aggregation. Interestingly, despite the strong adherence, RBC preserved their intact shape.

The ability of RBC to deform and the degree of aggregation are critical for RBC capillary passage and oxygen delivery to biotissues. Reduced RBC deformability contributes to the pathogenesis of various hematological disorders that impair the flow and augment RBC retention in the spleen. Expectedly, the attachment of NP onto RBC membrane increases the RBC stiffness. Decreased RBC deformability was previously reported for PEGylated Au NP [39] and Ag NP [21]. The distribution of NP on RBC surface was quite uneven, including NP agglomerates. To perform an adequate NP/RBC loading ratio [17], NP must be functionalized to increase their stability. In the present study, only highly perturbed spiculated RBC (EII) demonstrated a significant loss of elasticity. Since aggregation force and relative elongation measured for echinocytes for NP-treated samples were not different from the control, the nature of this shape transition probably was not caused by the direct interaction with NP. SEM observations demonstrated how NP interaction with RBC causes local membrane distortions, which subsequently can change the RBC surface tension and result in increased rigidity.

The aggregation of intact RBC was increased for all the NP-treated samples except Ag NP at 50 μg mL${}^{-1}$. NP attached to RBC can change the surface’s charge distribution and either facilitate the interaction or promote the repulsive mechanisms. In the current investigation, the attachment of NP to RBC appears to be non-specific, as both the NP and the RBC surface have a negative ζ-potential (Supplementary Materials, Table S1), which suggests electrostatic repulsion. In agreement with previous study [40], we suggest that the low degree of RBC shape deterioration, corresponding to EI can be easily tolerated by the microvasculature. Significant reduction aggregation for EII could be a result of the limited elasticity and spiculated surface hindering the formation of the contact surface area large enough to form a stable aggregate, which is in vivo conditions can lead to increased blood viscosity [41].

When introduced into the bloodstream, NP are expected to be covered by plasma proteins (protein corona), which can significantly modify the intrinsic properties of NP in unpredictable ways. All NP preserved their absorption properties in blood plasma (see Figure 5b), therefore, it could be suggested that blood proteins do not affect the optical properties of NP. There was no change in absorption at 1064 nm wavelength used for the optical trapping of RBC.

Plasmonic NP can convert light into heat, which is applied in photothermal therapy (PTT), aiming for the destruction of cancer cells under NIR light. NP being in the focal area of the OT can be heated to extremely high temperatures reaching hundreds of celsius degrees [42]. The average time required for RBC to burst is clearly shorter for TiN and ZrN NP (up to 52 s) at the same trap power (40 mW, 13 pN) compared with Au and Ag NP with a time scale upper range of about 100 s (see Figure 5d). No significant difference in the size of NP aggregates was found, however, the distribution of Ag NP indicates a higher fraction of smaller agglomerates (see Figure 5e). However, the times of RBC destruction for TiN and ZrN NP were significantly shorter than in the case of Ag and Au NP (see Figure 5f). Indeed, the plasmonic peaks of TiN NP and ZrN NP are red-shifted compared to their counterparts, having a tail beyond 800 nm, which is the reason for higher photothermal conversion efficiency. In accordance with conclusions made in the study [30], we suggest that this effect is a result of the higher plasmonic efficiency of TiN and ZrN NP compared to Au NP and Ag NP. Laser heating of the RBC without structural changes is possible in the range of 37–70 °C, and spherocyte formation with following rupture can happen in the range of 70–94 °C [43]. Controlled application of laser irradiation can be used for optoporation of RBC for intracellular delivery. Observed earlier off-resonant excitation by the IR optical trap by authors of [44] probably explains the significant temperature increase enough to cause hyperthermic RBC destruction of RBC membrane.

The approach in this study includes a minimal set of hemotoxicity risk assessment to satisfy before performing in vivo studies with intravascular administration of NP. It is worth noting that interpretation of in vitro results is limited due to the extra in vivo factors such as dynamic interactions with blood contents (protein corona formation, activation of platelets and white blood cells) and shear flow of the bloodstream. Interaction of NP with immune cells and platelets is likely to initiate a cascade of immune and thrombolytic reactions. Protein corona formation and opsonization of NP will decrease the circulation time and increase their clearance from the body.

The key strategies developed in recent decades explored the advantages of using RBC as a drug delivery platform. The attachment of therapeutics to RBC by specific antibody-mediated [45] or non-specific coupling may adversely affect RBC properties [17,46]. Among the achieved benefits of therapeutic conjugation with RBC are prolonged circulation [47], enhanced activity of the enzymes which substrates circulate in plasma [48], in vivo targeting of RBC from plasma [49], and delivery to the specific site of therapeutic interest such as blood clots [50,51] and vessel wall [52,53]. Further research should be focused on the functionalization of tested NP to improve their stability and provide homogeneous loading onto RBC, and elucidation of the mechanisms behind NP-RBC interaction to ensure beneficial conjugation.

## 5. Conclusions

The in vitro testing of cellular parameters serves as the first safety evaluation step of newly developed laser-synthesized plasmonic NP for biomedical applications. In this study, the hemorheological properties of RBC were examined via various microscopy methods and OT after incubation with plasmonic NPs synthesized by LAL. Plasmonic NP demonstrated hemolysis for concentrations exceeding 100 μg mL${}^{-1}$, while at the non-hemolytic range 50 μg mL${}^{-1}$ poikilocytosis and alterations in microrheological RBC properties as aggregation and deformability were found. The kinetics of RBC aggregation for RBC ensembles were found to be significantly different for samples treated with TiN and ZrN NP. This difference is a result of strong RBC adherence to the glass base of the chamber, which was identified using OT. Except for Ag NPs, increased RBC aggregation was observed after treatment with all tested NPs. At the same time, the stiffening of RBCs was demonstrated only for the highly perturbed echinocytes. The irradiated clusters of TiN and ZrN NPs are effective photothermal heat transducers, leading to a faster RBC hyperthermal haemolysis than Au and Ag NPs. Future research towards the development of NP-RBC conjugates for clinical applications should address proper NP functionalization to minimize NP-related RBC alterations, elucidation of the molecular mechanism behind NP interaction with RBC, and effects arising in whole blood in vivo.

## Data Availability

Data available upon request from the authors.

## Supplementary Materials

The following supporting information can be downloaded at: https://www.mdpi.com/article/10.3390/pharmaceutics15041046/s1, Figure S1: Colored SEM images of RBC treated with (a) Ag NP, (b) Au NP, (c) TiN NP, (d) ZrN NP at concentration 100 μg mL${}^{-1}$; Table S1: Size, zeta potential and polydispersity index (PdI) values (mean ± SD, n = 3) measured in deionized water (DI) and Dulbecco’s Phosphate Buffered Saline (DPBS); Table S2: Temporal aggregation parameters derived from the kinetics curves of the area occupied by RBC change in time.

## Abbreviations

The following abbreviations are used in this manuscript:

NP	Nanoparticles
RBC	Red blood cells
LAL	Laser ablation in liquid
OT	Optical tweezers
