# Supplementary material for: Impact of Plasmonic Nanoparticles on Poikilocytosis and Microrheological Properties of Erythrocytes
Source: Pharmaceutics. 2023 Mar 23;15(4):1046. doi: 10.3390/pharmaceutics15041046 (PMC10143243; doi:10.3390/pharmaceutics15041046)
Supplement: Supplementary file 1 [file pharmaceutics-15-01046-s001.zip › pharmaceutics-2223352-supplementary.pdf]

Supporting Information

Impact of plasmonic nanoparticles on hemorheology of erythrocytes

Tatiana Avsievich, Ruixue Zhu, Alexey P. Popov, Alexander Yatskovskiy, Anton A. Popov, Gleb Tikhonowsky, Andrei Pastukhov, Sergei Klimentov, Alexander Bykov, Andrei Kabashin and Igor Meglinski**

Table S1. Zeta potential values (mean ± SD, N = 3) measured in deionized water (DI) and Dulbecco's Phosphate Buffered Saline (DPBS).

Sample	Zeta Potential (mW)	
	DI	DPBS
Ag NP	-31.6 ± 3.3	-28.4 ± 1.6
Au NP	-33 ± 3.4	-21 ± 1.31
TiN NP	-38.7 ± 2.8	-20 ± 1.2
ZrN NP	-39.2 ± 0.9	-17.6 ± 1.2

The parameters to characterize RBC aggregation in syllectometry are usually retrieved from the exponential representation of kinetics curves. The kinetics of aggregation process in static conditions was retrieved from the optical microscopy images by calculating the change of area occupied by RBCs:

$$S_{RBCs}(t) = a \cdot exp^{\left(-\frac{x}{t_1}\right)} + c,$$

within 2 h (see Fig. 3 (a)). Aggregation kinetics is characterized by a time constant t_1 and by a half time $T_{1/2}$, which is the time interval where the area occupied by RBC is reduced by half the amplitude a.

Table S2. Temporal aggregation parameters derived from the kinetics curves of the area occupied by RBC change in time.

Sample	t_1, min	$T_{1/2}$, min
Control	23.8 ± 1.1	16.5 ± 0.8
Ag NP	29.4 ± 1.4	20.4 ± 0.9
Au NP	28.3 ± 1	19.6 ± 0.7
TiN NP	119.34 ± 16.9	82.7 ± 11.7
ZrN NP	13.2 ± 1.3	9.2 ± 0.9

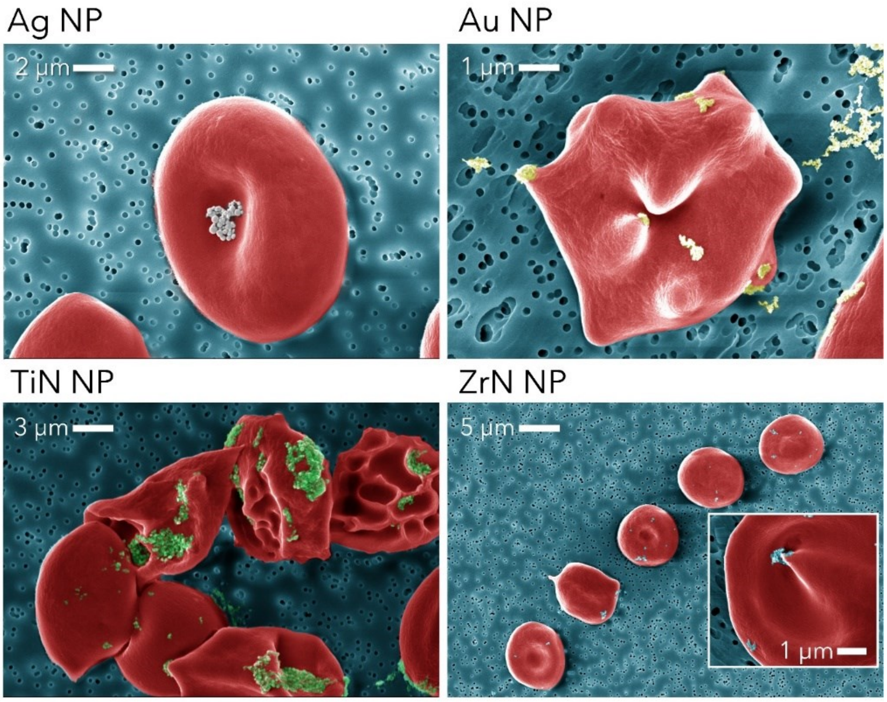

Figure S1. Colored SEM images of RBC treated with (a) Ag NP, (b) Au NP, (c) TiN NP, (d) ZrN NP at concentration 100 µg mL^{-1}.
